# Impact of a structured patient engagement program on treatment adherence, injection persistence, and visual outcomes in real-world anti-VEGF therapy for neovascular age-related macular degeneration

**DOI:** 10.1186/s12886-026-05045-8

**Published:** 2026-06-20

**Authors:** Lixia Lan, Jianhua Lan, Xuan Ren, Bo Ye

**Affiliations:** 1 Department of Fundus, Nanchang Aier Eye Hospital, Nanchang, 330000 China; 2 Department of Surgery, Gao’an People’s Hospital, Gao’an, 330800 China

**Keywords:** Neovascular age-related macular degeneration, Anti-VEGF therapy, Treatment adherence, Injection persistence, Patient engagement, Visual outcomes

## Abstract

**Background:**

Neovascular age-related macular degeneration (nAMD) is a leading cause of irreversible visual impairment among older adults. Although intravitreal anti–vascular endothelial growth factor (anti-VEGF) therapy has improved visual outcomes, real-world effectiveness is often limited by poor adherence and treatment discontinuation. This study evaluated the impact of a structured patient engagement program on treatment adherence, injection persistence, visual outcomes, and patient-reported outcomes in patients receiving anti-VEGF therapy for nAMD.

**Methods:**

This single-center retrospective observational comparative cohort study included 350 patients with nAMD treated between January 2022 and December 2024. Patients received either standard care alone (*n* = 175) or standard care plus a structured patient engagement program (*n* = 175) incorporating individualized education, adherence counseling, appointment reminders, and proactive follow-up communication. Primary outcomes were treatment adherence and injection persistence. Secondary outcomes included best-corrected visual acuity (BCVA), patient satisfaction, and disease understanding.

**Results:**

Patients enrolled in the engagement program demonstrated significantly better adherence than those receiving standard care. The mean number of missed injection visits was lower (0.51 ± 0.68 vs. 1.48 ± 1.27; *P* < 0.001), and delayed injections exceeding two weeks were less frequent (22.3% vs. 50.9%; *P* < 0.001). Good adherence was achieved in 89.1% of patients in the engagement group compared with 50.3% in the standard care group (*P* < 0.001), while treatment persistence was higher (90.9% vs. 77.7%; *P* = 0.001). Final BCVA was significantly better in the engagement group (0.61 ± 0.30 vs. 0.70 ± 0.34 LogMAR; *P* = 0.015), and a greater proportion of patients experienced visual improvement (65.1% vs. 52.6%; *P* = 0.023). Patient satisfaction and disease understanding were also significantly higher among patients receiving the engagement intervention (*P* < 0.001 for both). Safety outcomes were comparable between groups.

**Conclusions:**

A structured patient engagement program was associated with improved treatment adherence, enhanced injection persistence, and better short- to mid-term visual outcomes in patients receiving anti-VEGF therapy for nAMD. These findings support the integration of patient-centered engagement strategies into routine retinal care to optimize real-world treatment outcomes.

## Introduction

Age-related macular degeneration (AMD) is a chronic retinal disease that is considered to be one of the major causes of irreversible blindness in the elderly population [1, 2]. In recent years, with the rapid progression of the world population’s aging process, AMD has been considered to be a major cause of public health and socioeconomic problems [3, 4]. AMD is a disease that primarily affects the macula, leading to the deterioration of central vision [5, 6]. Central vision is considered to be critical for performing various tasks like driving a vehicle or recognizing faces [7]. Among the two forms of AMD that have been identified, the majority of cases of irreversible blindness associated with AMD have been attributed to neovascular or “wet” AMD [8]. Despite being responsible for fewer cases of AMD compared to its counterpart, “wet” AMD accounts for the majority of cases of irreversible blindness associated with AMD. “Wet” AMD is considered to be a form of AMD that results in abnormal choroidal neovascularization in the retina [9]. Such abnormal neovascularization results in the leakage of blood in the retina, leading to the formation of scars that can cause a quick deterioration in vision [8].

Over the last two decades, the introduction of intravitreal anti-vascular endothelial growth factor (anti-VEGF) therapy has revolutionized the treatment of “wet” AMD [10]. Such therapy has been considered to be the standard treatment for “wet” AMD. Inhibitors of VEGF like ranibizumab, Conbercept, and aflibercept, have been used to treat “wet” AMD [11–13]. Such inhibitors have been considered to be effective in the suppression of abnormal choroidal neovascularization in the retina. Such suppression results in the improvement of vision in patients with “wet” AMD. Large clinical studies have shown that injections of anti-VEGF inhibitors can be used to improve or preserve vision in patients with “wet” AMD [13, 14].

Although favorable results have been documented in clinical trials, evidence is growing that in real-world conditions, outcomes may not compare favorably to those documented in clinical trials. Indeed, in real-world conditions, patients may not adhere to recommended injection regimens, which may result in undertreatment and reduced visual acuity gains [15, 16]. The challenges may arise from a range of factors, including patient age, the burden of repeated clinic visits, inadequate patient understanding of disease progression, and healthcare access issues [17]. The gap in efficacy and real-world outcomes remains a major concern in the long-term management of neovascular AMD.

However, in the last few years, more emphasis has been placed on patient-centered interventions to improve adherence to chronic eye diseases, such as those requiring repeated intravitreal injections. Indeed, patient engagement has been described as a potential solution to addressing the problem of non-adherence to eye treatments. This may involve interventions such as patient education, regular communication, counseling, and reminder interventions, among others [18, 19]. This is particularly true in conditions such as neovascular AMD, where repeated intravitreal injections may be required [20, 21]. Indeed, patient education may help patients become more aware of disease progression and the benefits of treatment, thus improving adherence to treatment regimens and minimizing the risk of discontinuation [21].

Research in the management of chronic diseases has shown that patient education and support interventions can lead to improved patient compliance and patient satisfaction, thus leading to improved patient outcomes [22]. Similarly, in the field of ophthalmology, some emerging data suggest that counseling and follow-up interventions may help improve patient compliance and adherence to anti-VEGF therapy, although the data are limited, and most existing research has primarily addressed patient satisfaction and knowledge, not comprehensive patient outcomes [19, 22, 23].

In particular, there is a relative lack of real-world evidence evaluating whether structured patient engagement programs can simultaneously influence treatment adherence, injection persistence, and visual outcomes in patients receiving anti-VEGF therapy for neovascular AMD.

Given these considerations, further investigation is needed to better understand how patient-centered interventions may improve the effectiveness of anti-VEGF therapy in routine clinical practice. Therefore, the present study aimed to evaluate the impact of a structured patient engagement program on treatment adherence, injection persistence, and visual outcomes among patients undergoing real-world anti-VEGF therapy for neovascular age-related macular degeneration.

## Materials and methods

### Study design and setting

This real-world comparative cohort study was conducted at the Department of Fundus, Nanchang Aier Eye Hospital, a tertiary ophthalmology center specializing in retinal diseases. The study aimed to evaluate the impact of a structured patient engagement program on treatment adherence, injection persistence, and visual outcomes among patients undergoing intravitreal anti–vascular endothelial growth factor (anti-VEGF) therapy for neovascular age-related macular degeneration (nAMD).

The study included patients treated between January 2022 and December 2024 as part of routine clinical practice. During this period, all eligible consecutive patients diagnosed with nAMD and receiving anti-VEGF therapy at the study center were screened for inclusion. A total of 350 patients who met the predefined eligibility criteria and had adequate clinical follow-up were included in the final analysis.

### Study population

Patients diagnosed with neovascular (wet) age-related macular degeneration who initiated anti-VEGF therapy at the study center during the study period were considered for inclusion. The diagnosis of nAMD was established based on comprehensive ophthalmic evaluation supported by multimodal retinal imaging, including dilated fundus examination and optical coherence tomography (OCT), consistent with established clinical diagnostic criteria.

Eligible participants were adults aged 50 years or older with active neovascular AMD requiring intravitreal anti-VEGF therapy and with available baseline clinical data and follow-up information. Only one eye per patient was included in the analysis to avoid intra-patient correlation. In cases where both eyes were eligible, the first treated eye was selected as the study eye. Patients were excluded if they had non-AMD causes of choroidal neovascularization, including myopic choroidal neovascularization or polypoidal choroidal vasculopathy, or other retinal diseases that could significantly affect visual outcomes, such as diabetic retinopathy or retinal vein occlusion. Patients with advanced glaucoma, visually significant cataract, retinal dystrophies, previous major ocular surgery during the study period, incomplete clinical records, or inadequate follow-up were also excluded. Patients who met the inclusion criteria were divided into categories based on whether they received standard anti-VEGF treatment alone or in conjunction with the structured patient engagement program.

### Anti-VEGF treatment protocol

All the patients underwent intravitreal anti-VEGF treatment, which is a routine clinical protocol adopted by the study center. The anti-VEGF drugs used included ranibizumab, aflibercept, and conbercept, which were used depending on the discretion of the treating physicians, the availability of the drugs, and other patient-specific factors.

The intravitreal injections were administered under aseptic conditions in a special procedure room by experienced ophthalmologists. Before the procedure, topical anesthesia and povidone-iodine antisepsis were used according to the clinical protocol.

The patients underwent the anti-VEGF treatment protocol, which included an initial loading dose with three monthly injections, followed by continued treatment depending on the activity of the disease, which was monitored during the follow-up period.

### Structured patient engagement program

In addition to routine physician counseling provided during standard clinical care, patients enrolled in the structured engagement program participated in a formalized adherence-support intervention designed to improve treatment understanding and persistence. The intervention was delivered by trained clinical coordinators and nursing staff under the supervision of treating ophthalmologists and included individualized education regarding neovascular AMD and anti-VEGF therapy, adherence-focused counseling, scheduled appointment reminder telephone calls, and proactive follow-up communication to identify barriers, address patient concerns, and reinforce treatment continuity. The components of the program were standardized across all participants to ensure consistency in content and delivery. Collectively, these measures were intended to enhance patient awareness, strengthen adherence, and minimize avoidable treatment interruption.

### Outcome measures

#### Primary outcomes

The primary outcomes of this study were treatment adherence and injection persistence. Treatment adherence was evaluated at the patient level over the defined observation period (minimum 6 months and up to 12 months). The expected number of visits and injections per patient was determined based on the standard treatment protocol, including an initial loading phase followed by maintenance therapy. “Good adherence” was defined as attendance at ≥ 80% of scheduled injection visits without delays exceeding two weeks beyond the planned treatment interval. The ≥ 80% threshold was selected based on commonly accepted adherence criteria used in chronic disease management and medication adherence research, where adherence rates of 80% or greater are generally considered indicative of satisfactory treatment compliance [23]. In addition, adherence and persistence have been recognized as important determinants of real-world outcomes in anti-VEGF therapy [17, 24]. Missed visits and delayed injections were recorded and incorporated into adherence assessment.

Treatment persistence was defined as the continuation of anti-VEGF therapy without permanent discontinuation during the observation period. Patients were classified as non-persistent if they discontinued treatment prematurely or experienced a prolonged unplanned interruption exceeding 3 months. Patients lost to follow-up were considered non-persistent.

#### Secondary outcomes

The secondary outcomes targeted in this study were the patients’ visual outcomes, which were measured using best-corrected visual acuity (BCVA). BCVA measurements were taken using routine eye examinations.

### Data collection

The data was collected from the hospital’s electronic medical records and institutional clinical database. The data variables included patients’ age, gender, baseline ocular characteristics, type and dosage of anti-VEGF agent, number of patients injected, number of visits, adherence, and visual acuity.

### Statistical analysis

Statistical analysis was carried out using SPSS software (version 22.0, IBM Corp., USA). Data were expressed as mean ± standard deviation for continuous variables and as counts and percentages for categorical variables. Statistical comparisons were performed using appropriate parametric and non-parametric tests. Results are presented with corresponding P-values, and findings are interpreted in the context of clinical relevance. Comparisons of these two kinds of variables between different groups were carried out using an independent t-test and chi-square test, respectively. A two-tailed P value less than 0.05 was considered statistically significant.

### Ethical considerations

This study was approved by the Ethics Committee of Nanchang Aier Eye Hospital (No. 2021(004)). All procedures were performed in accordance with the ethical standards laid down in the Declaration of Helsinki. Informed consent was obtained from all participants before any treatment and inclusion of patients in the clinical database.

## Results

### Study population

A total of 350 patients with neovascular age-related macular degeneration (nAMD) receiving intravitreal anti-vascular endothelial growth factor (anti-VEGF) therapy were included in the study. The cohort was evenly divided between the two study groups, with 175 patients receiving standard care and 175 patients participating in the structured patient engagement program.

The mean age of the study population was 71.16 ± 6.68 years (range: 55–88 years). Overall, 52.6% of patients were male, and 56.3% resided in urban areas. Approximately 32% of patients reported a history of smoking. The most common type of choroidal neovascularization was Type 1 CNV (60.0%), followed by Type 2 (24.9%) and mixed-type lesions (15.1%). Regarding treatment agents, ranibizumab was the most frequently used anti-VEGF drug (45.1%), followed by aflibercept (34.3%) and conbercept (20.6%).

At baseline, the mean best-corrected visual acuity (BCVA) was 0.79 ± 0.29 LogMAR, and the mean central retinal thickness (CRT) was 416.19 ± 75.77 μm, reflecting active neovascular disease.

### Baseline characteristics

Baseline demographic and clinical characteristics of the two study groups are summarized in Table 1. There were no statistically significant differences between the standard care and engagement program groups in terms of age, baseline BCVA, or central retinal thickness (all *P* > 0.05). Similarly, the distribution of gender, residence, educational attainment, CNV subtype, and anti-VEGF agents used did not differ significantly between groups (Table 1).

**Table 1:** Baseline demographic and clinical characteristics of the study population

Variable	Standard Care (*n* = 175)	Engagement Program (*n* = 175)	*P*-value
Age (years)	71.40 ± 6.88	70.93 ± 6.49	0.508
Baseline BCVA (LogMAR)	0.82 ± 0.31	0.77 ± 0.28	0.111
Central Retinal Thickness (µm)	415.61 ± 74.41	416.77 ± 77.31	0.887
Male, n (%)	89 (50.9%)	95 (54.3%)	0.592
Female, n (%)	86 (49.1%)	80 (45.7%)	
Urban residence, n (%)	92 (52.6%)	105 (60.0%)	0.196
Rural residence, n (%)	83 (47.4%)	70 (40.0%)	
Education Level			0.219
Primary	77 (44.0%)	64 (36.6%)	
Secondary	56 (32.0%)	64 (36.6%)	
Higher	20 (11.4%)	28 (16.0%)	
Smoker, n (%)	68 (38.9%)	44 (25.1%)	0.008
Non-smoker, n (%)	107 (61.1%)	131 (74.9%)	
CNV Type1, n (%)	109 (62.3%)	101 (57.7%)	0.644
CNV Type2, n (%)	42 (24.0%)	45 (25.7%)	
CNV Mixed, n (%)	24 (13.7%)	29 (16.6%)	0.380
Ranibizumab, n (%)	73 (41.7%)	85 (48.6%)	
Aflibercept, n (%)	62 (35.4%)	58 (33.1%)	
Conbercept, n (%)	40 (22.9%)	32 (18.3%)	

Values are presented as mean ± standard deviation or number (%). P-values were calculated using independent t-test for continuous variables and chi-square tests, for categorical variables

A statistically significant difference was observed in smoking status between the groups (*P* = 0.008), as shown in Table 1. Although baseline demographic and clinical characteristics were generally comparable between groups, smoking prevalence was significantly higher in the standard care group (38.9% vs. 25.1%, *P* = 0.008), representing a potential confounding variable that should be considered when interpreting comparative outcomes.

### Treatment characteristics

Treatment characteristics are presented in Table 2. The mean number of intravitreal injections during the study period was 6.30 ± 2.00 in the standard care group and 5.81 ± 2.15 in the engagement program group. This difference was statistically significant (*P* = 0.026), as shown in Table 2.

**Table 2:** Treatment characteristics of the study population

Variable	Standard Care (*n* = 175)	Engagement Program (*n* = 175)	*P*-value
Total number of injections per patient	6.30 ± 2.00	5.81 ± 2.15	0.026
Mean injection interval (weeks)	5.90 ± 1.18	5.85 ± 1.11	0.705
Duration of treatment (months)	9.23 ± 2.04	8.99 ± 2.01	0.268

Nevertheless, there were no major variations in the average injection interval and overall treatment duration between the two groups (*P* > 0.05 for both variables), suggesting that the treatment regimen for both groups was relatively similar (Table 2).

### Treatment adherence and injection persistence

Table 3 provides an overview of the results for the outcome variables for treatment adherence and injection persistence. The results indicate that patients in the structured engagement program showed better adherence to treatment compared to those in the standard care group.

The results in Table 3 show that the average number of missed injection visits for patients in the engagement program (0.51 ± 0.68) was significantly lower compared to the standard care group (1.48 ± 1.27; *P* < 0.001). In addition, delayed injections for more than two weeks were substantially fewer in the engagement program group (22.3%) compared to the standard care group (50.9%), as shown in Table 3.

**Table 3:** Adherence and persistence outcomes of the study population

Outcome	Standard Care (*n* = 175)	Engagement Program (*n* = 175)	*P*-value
Number of missed injection visits per patient	1.48 ± 1.27	0.51 ± 0.68	< 0.001
Delayed injections > 2 weeks, n (%)	89 (50.9%)	39 (22.3%)	< 0.001
Good adherence, n (%)	88 (50.3%)	156 (89.1%)	< 0.001
Treatment persistence, n (%)	136 (77.7%)	159 (90.9%)	0.001

Values are presented as mean ± standard deviation or number (%). P-values were calculated using independent t-test or chi-square tests, as appropriate

The absolute difference in good adherence between the engagement and standard care groups was 38.9% (95% CI: 30.1%–47.6%), while the difference in treatment persistence was 13.1% (95% CI: 5.6%–20.6%)

Good treatment adherence was observed in 89.1% of patients in the engagement program group compared with 50.3% in the standard care group (*P* < 0.001). Additionally, treatment persistence was significantly higher in the engagement program group (90.9% vs. 77.7%, *P* = 0.001), as summarized in Table 3.

**Fig. 1:**
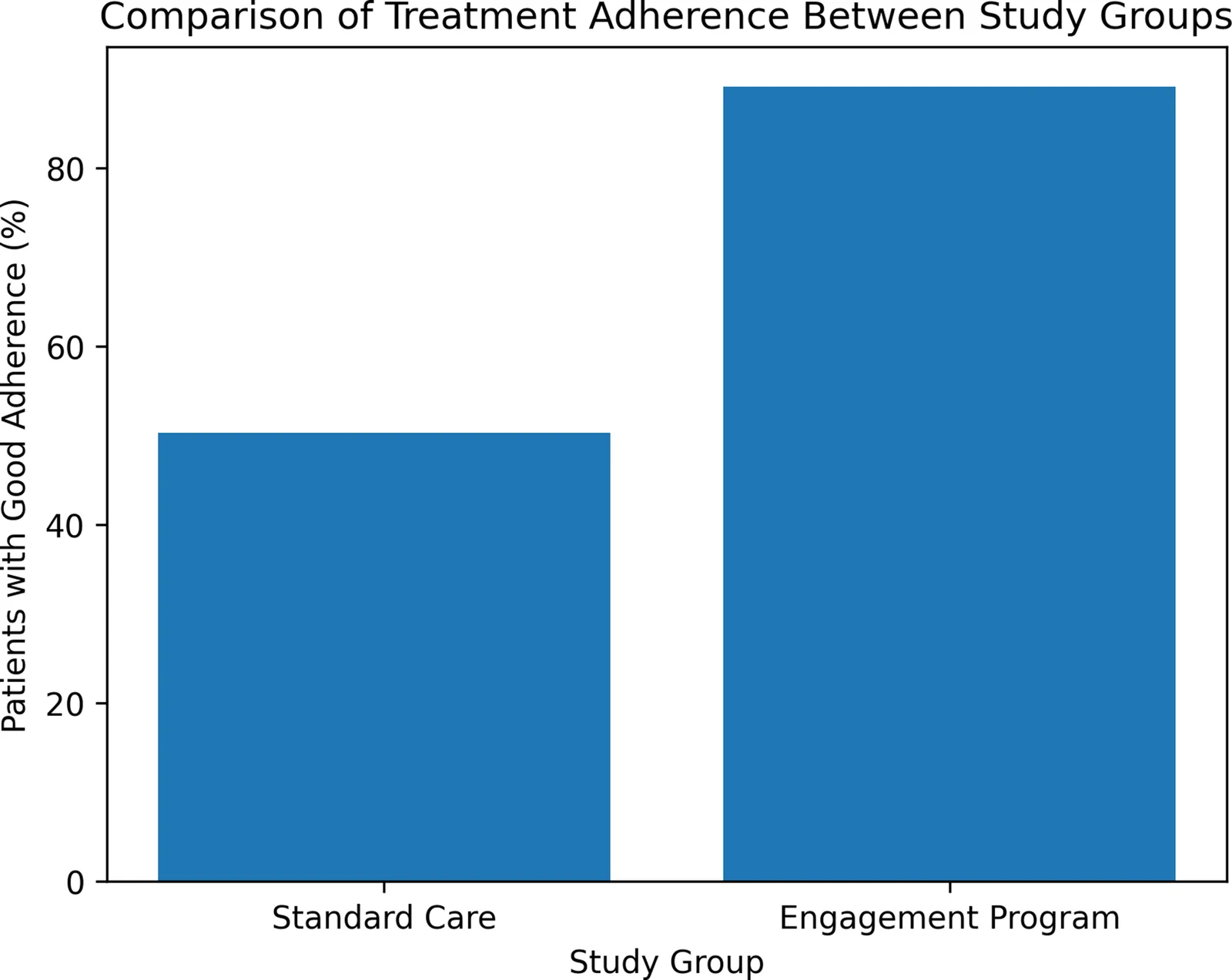
Comparison of treatment adherence between patients receiving standard care and those enrolled in the structured patient engagement program during anti-VEGF therapy for neovascular age-related macular degeneration. A higher proportion of patients in the engagement program demonstrated good adherence to treatment

The difference in adherence rates between the two groups is illustrated in Fig. 1, demonstrating a substantially higher proportion of patients with good adherence in the engagement program group compared with the standard care group.

### Visual outcomes

Visual outcomes are presented in Table 4. Baseline BCVA did not differ significantly between the two groups (0.82 ± 0.31 vs. 0.77 ± 0.28 LogMAR, *P* = 0.111), confirming comparable baseline visual status (Table 4).

**Table 4:** Visual outcomes of the study population

Variable	Standard Care (*n* = 175)	Engagement Program (*n* = 175)	*P*-value
Baseline BCVA (LogMAR)	0.82 ± 0.31	0.77 ± 0.28	0.111
Final BCVA (LogMAR)	0.70 ± 0.34	0.61 ± 0.30	0.015
Change in BCVA (LogMAR)	0.12 ± 0.16	0.16 ± 0.16	0.050
Patients with visual improvement, n (%)	92 (52.6%)	114 (65.1%)	0.023

Values are presented as mean ± standard deviation or number (%). P-values were calculated using independent t-test or chi-square tests, as appropriate. Positive values for BCVA change indicate improvement (reduction in LogMAR)

The absolute difference in the proportion of patients achieving visual improvement between groups was 12.6% (95% CI: 2.3%–22.8%). The mean between-group difference in BCVA change was 0.033 LogMAR (95% CI: 0.000–0.067)

At the final follow-up visit, however, patients in the engagement program demonstrated significantly better visual acuity compared with those receiving standard care (0.61 ± 0.30 vs. 0.70 ± 0.34 LogMAR, *P* = 0.015), as shown in Table 4. The mean improvement in BCVA was also greater in the engagement program group (0.16 ± 0.16 LogMAR) compared with the standard care group (0.12 ± 0.16 LogMAR), reaching borderline statistical significance (*P* = 0.050) (Table 4).

Furthermore, a significantly higher number of patients receiving care through the engagement program showed signs of improvement in terms of visual acuity compared with patients receiving routine care (65.1% vs. 52.6%, *P* = 0.023), as shown in Table 4.

**Fig. 2:**
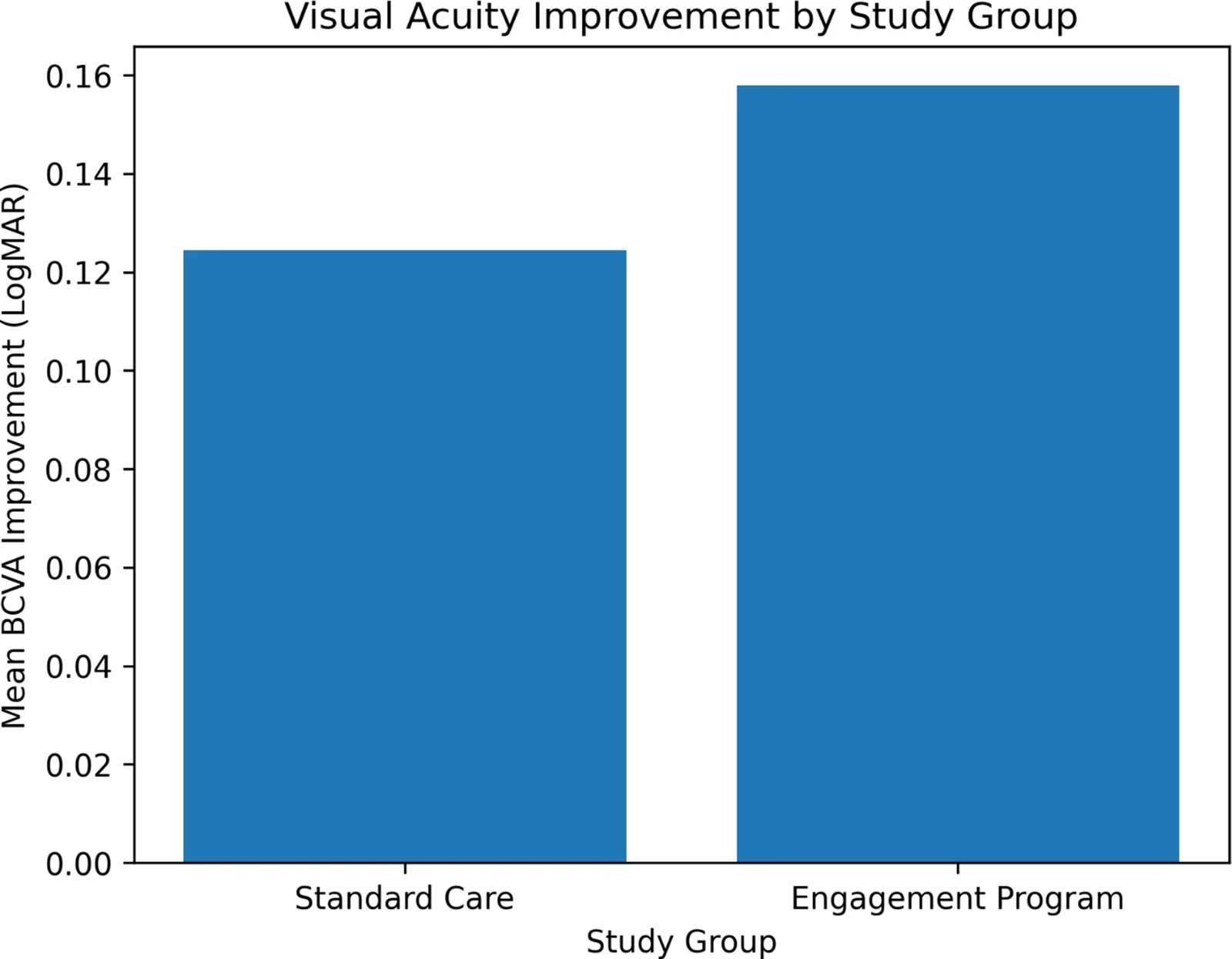
Mean improvement in best-corrected visual acuity (BCVA) in patients receiving standard care compared with those enrolled in the structured patient engagement program during anti-VEGF therapy for neovascular age-related macular degeneration

The improvement in visual acuity of patients between the two care approaches is shown in Fig. 2, which illustrates a higher improvement in terms of BCVA for patients receiving care through the structured patient engagement program.

### Patient-reported outcomes

Patient-reported outcomes are summarized in Table 5. The structured patient engagement program was associated with substantially higher levels of patient satisfaction.

In the engagement program group, 56.0% of patients reported being very satisfied with their treatment, compared with 30.9% in the standard care group (*P* < 0.001). Conversely, dissatisfaction with treatment was markedly lower among patients receiving the engagement program (7.4% vs. 29.1%), as shown in Table 5.

**Table 5:** Patient‑reported outcomes of the study population

Outcome	Standard Care (*n* = 175)	Engagement Program (*n* = 175)	*P*-value
Very satisfied, n (%)	54 (30.9%)	98 (56.0%)	< 0.001
Satisfied, n (%)	70 (40.0%)	64 (36.6%)	
Not satisfied, n (%)	51 (29.1%)	13 (7.4%)	
Good disease understanding, n (%)	57 (32.6%)	119 (68.0%)	< 0.001

Values are presented as number (%). P-values were calculated using chi-square test

Disease understanding was also significantly improved among patients enrolled in the engagement program. Good understanding of the disease and treatment was reported by 68.0% of patients in the engagement group, compared with 32.6% in the standard care group (*P* < 0.001) (Table 5).

### Safety outcomes

Safety outcomes are presented in Table 6. Overall, intravitreal anti-VEGF therapy was well tolerated, with a low incidence of treatment-related complications.

**Table 6:** Safety outcomes of the study population

Outcome	Standard Care (*n* = 175)	Engagement Program (*n* = 175)	*P*-value
Endophthalmitis, n (%)	1 (0.6%)	2 (1.1%)	1.000
Increased IOP, n (%)	7 (4.0%)	7 (4.0%)	1.000
Retinal detachment, n (%)	0 (0.0%)	0 (0.0%)	—

Values are presented as number (%). P-values were calculated using chi-square or Fisher’s exact test as appropriate. P-value was not calculated for outcomes with zero events in both groups. Safety events are presented on a per-patient basis

Endophthalmitis occurred in three patients during the study period. Because complication rates were reported on a per-patient basis rather than a per-injection basis, the apparent incidence may appear higher than conventionally reported per-injection rates in the literature. No statistically significant difference was observed between groups (*P* = 1.000), as shown in Table 6. Transient increases in intraocular pressure were observed in 4% of patients in each group, while no cases of retinal detachment were reported during the study period (Table 6).

## Discussion

### Overview of key findings

In this real-world comparative cohort study conducted at a tertiary retinal center, we observed that implementation of a structured patient engagement program was associated with significant improvements in treatment adherence, injection persistence, and visual outcomes among patients receiving intravitreal anti-VEGF therapy for neovascular AMD. Compared with the standard care alone, patients in the engagement program had reduced missed injection visits, reduced percentages of delayed injections of more than two weeks, increased percentages of good adherence, and significantly increased treatment persistence. Importantly, these differences in treatment behavior were accompanied by superior functional outcomes, including final BCVA and percentages of patients showing visual improvement. At the same time, patient-reported patient satisfaction and disease understanding were dramatically improved in the engagement program, while safety was equivalent in the two groups. Educational attainment was also comparable between groups at baseline, reducing the likelihood that differential educational status substantially influenced observed adherence differences. Overall, these data suggest that patient-level interventions can have significant effects on treatment behavior and effectiveness in routine anti-VEGF therapy.

### Interpretation of adherence and persistence results

Adherence and persistence are essential components of treatment effectiveness in chronic intravitreal therapy. Our data show that, compared with the structured engagement program, patients receiving only standard care had significantly more missed visits and greater percentages of delayed injections of more than two weeks. However, almost 90% of patients in the engagement program had good adherence, compared with only half of those receiving only standard care. Treatment persistence was also significantly increased in the engagement program.

Such findings emphasize the potential of structured education, counseling, reminders, and follow-up communications in improving treatment compliance and persistence. In this context, anti-VEGF treatment was found to be burdensome, particularly in elderly patients with mobility issues or other comorbidities. Without a clear understanding of the chronic nature of their disease and the consequences of nonadherence to treatment, patients may not appreciate the necessity of attending their scheduled appointments. In this regard, the engagement program may have helped patients overcome the above challenges by reinforcing the chronic nature of their disease, the necessity of repeated treatments, and minimizing other barriers through reminders and other support mechanisms. In real-world practice, patients do not adhere to their treatment regimens as recommended in clinical trials, and this can affect their treatment outcomes in a negative way [17, 24–26].

### Relationship between adherence and visual outcomes

The improvement in treatment adherence and persistence in the engagement group was accompanied by significantly improved visual outcomes. Although the baseline BCVA and central retinal thickness in both groups were comparable, patients in the engagement program had better final BCVA and a greater proportion of patients with visual improvement compared to the control group. In addition, BCVA improvement showed borderline statistical significance (*P* = 0.050).

In this context, it is logical to assume that the improved treatment adherence and persistence in the engagement group were accompanied by better visual outcomes because anti-VEGF treatment requires continuous suppression of VEGF to prevent the recurrence of choroidal neovascularization activity. It is also important to acknowledge that smoking prevalence was significantly higher in the standard care group at baseline. Given that smoking is a recognized risk factor for AMD progression and may negatively influence treatment response and visual prognosis, this imbalance should be considered when interpreting comparative visual outcomes. Any interruption in treatment or increased time between treatments can reactivate choroidal neovascularization, leading to recurrent exudation and hemorrhage and ultimately photoreceptor damage that can be irreversible in nature [27–29]. In this context, anti-VEGF treatment regimens in clinical trials have shown that continuous and protocol-driven anti-VEGF treatment regimens can maintain anatomical control and functional outcomes in patients with nAMD, whereas real-world studies have shown undertreatment and poor visual outcomes in patients with nAMD treated with anti-VEGF agents [30].

### Comparison with previous literature

Our results regarding the adherence and persistence rates in the standard care group are similar to those reported in the real-world data, which have demonstrated significant rates of missed appointments, treatment discontinuation, and delayed injections in patients with neovascular AMD. Some observational studies have reported persistence rates varying from 60% to 80% during the first year of treatment, with rates decreasing over time [28, 29]. Our persistence rates of 77.7% in the standard care group are consistent with these data.

On the other hand, the 90.9% persistence rates and high adherence rates observed in the engagement program group are higher than those reported in real-world data. Although there are limited studies on the effectiveness of structured patient engagement programs on patient adherence in patients with nAMD, there is emerging evidence on the effectiveness of reminder systems and counseling on reducing loss to follow-up and improving appointment attendance [17]. However, this study extends these data by showing not only improvements in adherence but also improvements in vision outcomes in a relatively large real-world patient population. These findings are consistent with previous real-world studies that have reported suboptimal adherence and persistence in patients receiving anti-VEGF therapy for neovascular AMD. Prior studies have demonstrated that missed visits and delayed injections are associated with poorer visual outcomes, highlighting the importance of adherence-support strategies. Our results extend this evidence by suggesting that structured patient engagement interventions may improve adherence and short- to mid-term outcomes in routine clinical practice.

It is interesting to note that despite having one fewer total injections, the engagement group had better visual outcomes. This could highlight the significance of timely and consistent delivery of injections rather than the total number of injections.

### Patient-centered outcomes

In addition to the clinical outcomes, it is evident that there were significant improvements in patient satisfaction and understanding of disease. More than half of the patients receiving the engagement intervention were “very satisfied,” and two-thirds had good understanding of disease, whereas these figures were considerably less for the standard care group.

Chronic eye diseases, such as neovascular age-related macular degeneration, require a partnership between doctor and patient over a long period of time. Education and communication may play a key role in empowering patients and developing a greater level of trust and motivation for adhering to arduous treatment regimens. Patients’ understanding of disease may reduce apprehension and uncertainty related to repeated injections [31–33].

In this regard, our study supports the growing movement towards a new model of patient-centered care for ophthalmology, recognizing communication as an integral part of therapeutic success rather than an ancillary benefit.

### Safety findings

Intravitreal anti-VEGF therapy was found to be well tolerated in both groups. Although endophthalmitis events were rare in absolute number, we acknowledge that reporting these complications on a per-patient rather than per-injection basis may yield numerically higher apparent rates than those typically reported in large injection-based safety studies. Importantly, complication rates did not differ significantly between groups, and no new safety concerns related to the engagement intervention were identified. These results are consistent with the known safety profile of anti-VEGF therapy, as demonstrated in large clinical trials and real-world studies [27, 28, 33].

### Clinical implications

The current research has several implications. Currently, bridging the gap between trial efficacy and real-world effectiveness is a major unmet need in the management of nAMD. Patient engagement programs might represent an effective solution to improve patient adherence without changing treatment paradigms. Patient education, reminder systems, and follow-up communication can be integrated into current treatment pathways through trained coordinators or nursing staff. This might have the potential to improve functional outcomes, thus maximizing the potential benefits of treatment while reducing the risk of vision loss.

### Strengths of the study

This research has several strengths. First, the real-world patient population consisted of 350 patients, which can be regarded as a large patient population. Second, the research assessed several parameters of treatment success. Third, the structured patient engagement intervention was incorporated into current treatment pathways, thus maximizing the real-world relevance of the research. It is important to emphasize that this study represents observational real-world evidence, and therefore causal inferences should be made with caution. Although associations between the engagement program and improved outcomes were observed, residual confounding cannot be fully excluded.

### Study limitations

There are some limitations of this study that need to be discussed. First of all, it is a single-center study and could be limited in its generalizability to different healthcare settings with different patient demographics and resources available. The potential for selection bias cannot be fully excluded as a result of this being an observational comparative cohort study design; however, as mentioned before, patient demographics were largely comparable between each group. The difference in smoking status between each group could be a potential source of residual confounding variables. Smoking is a known risk factor influencing disease progression in age-related macular degeneration and may potentially influence treatment outcomes; therefore, this imbalance should be considered when interpreting the results. The follow-up period of this study was relatively short-term, and long-term persistence and visual outcomes are yet to be determined. The absence of multivariable adjustment may limit the precision of effect estimation.

Although educational attainment was incorporated into baseline demographic assessment, this study did not specifically evaluate whether education level modified responsiveness to the structured engagement intervention, which may represent an important area for future investigation. Because this was a non-randomized observational study, selection bias and residual confounding cannot be fully excluded. As this was a single-center study, the generalizability of these findings to other settings and healthcare systems may be limited.

### Future research directions

Further studies are needed to validate these findings and assess long-term sustainability of such interventions in a multiple center design. Further randomized studies could also be carried out to establish cause and effect relationships between engagement interventions and patient outcomes. Cost-effectiveness studies could also be carried out to assess the economic implications of implementing such engagement programs in patients with neovascular AMD receiving anti-VEGF therapy. The integration of digital adherence aids, mobile health technologies, and telemedicine-based monitoring approaches could also be explored as potential areas of innovation in managing patients with chronic retinal diseases.

## Conclusion

In this real-world observational comparative cohort study, participation in a structured patient engagement program was associated with improved treatment adherence, greater injection persistence, and better short- to mid-term visual outcomes among patients with neovascular age-related macular degeneration receiving intravitreal anti-VEGF therapy. Patients enrolled in the engagement program also experienced fewer missed or delayed appointments compared with those receiving standard care alone.

Structured education and follow-up communication were further associated with improved disease understanding and higher patient satisfaction, highlighting the potential value of patient-centered strategies in the management of chronic retinal diseases.

Given the relatively limited follow-up duration and observational study design, these findings should be interpreted cautiously. Further prospective multicenter studies with longer follow-up are needed to confirm the durability of these associations and to better define the role of structured patient engagement in optimizing real-world anti-VEGF treatment outcomes.

## Data Availability

The datasets used and analyzed during the current study are available from the corresponding author on reasonable request.

## Abbreviations

AMD: Age-related macular degeneration
nAMD: Neovascular age-related macular degeneration
VEGF: Vascular endothelial growth factor
anti-VEGF: Anti–vascular endothelial growth factor
BCVA: Best-corrected visual acuity
CRT: Central retinal thickness
OCT: Optical coherence tomography
IOP: Intraocular pressure
CNV: Choroidal neovascularization
